# EVALI Vaping Liquids Part 2: Mass Spectrometric Identification of Diluents and Additives

**DOI:** 10.3389/fchem.2021.746480

**Published:** 2021-10-25

**Authors:** Laura A. Ciolino, Travis M. Falconer, Tracy L. Ranieri, Jana L. Brueggemeyer, Allison M. Taylor, Angela S. Mohrhaus

**Affiliations:** Forensic Chemistry Center, United States Food and Drug Administration, Cincinnati, OH, United States

**Keywords:** EVALI, vaping liquids, e-cigarettes, vitamin E acetate (VEA), diluents, additives, GC-MS, LC-HRMS

## Abstract

The vaping liquid additive vitamin E acetate (VEA) was strongly linked to the 2019 United States nationwide outbreak of pulmonary lung illness (EVALI) associated with e-cigarettes or vaping liquids. Our laboratory received over 1,000 vaping liquid products for identification of the vaping liquid additives, including hundreds of vaping products from EVALI patients. In this work, we present results obtained for the GC-MS identification of numerous vaping liquid additives in a large subset of ca. 300 Cannabis vaping liquids, including vitamin E acetate, medium chain triglycerides oil (MCT oil), polyethylene glycols, squalane, triethyl citrate, dipropylene glycol dibenzoate (DPG dibenzoate), pine rosin acids, pine rosin methyl esters, and sucrose acetate isobutyrate (SAIB). Confirmation of DPG dibenzoate and SAIB using LC-HRMS is also presented. GC-MS analysis for additives identified as the parent compounds was conducted after separation on a commercial 5% phenyl phase. GC-MS analysis for additives identified as the trimethylsilyl derivatives was conducted after separation on a commercial 35% silphenylene phase. LC-HRMS analysis was conducted using gradient elution with either C18 or phenyl-hexyl phases and determination of exact masses for the target compounds. In addition to providing rapid methods for the identification of vaping liquid additives, this work highlights the variety of Cannabis vaping liquid additives in current use.

## Introduction

In Part 1 (Ciolino et al., 2021), we presented results obtained for the gas chromatography-mass spectrometry (GC-MS) analysis of a series of THC isomers in THC-vaping liquids associated with the 2019 nationwide outbreak of pulmonary lung illness (EVALI). The vaping liquids from Part 1 were a large subset (ca. 300 vapes) of over 1,000 Cannabis vaping liquids which were analyzed by our laboratory in conjunction with the EVALI health crisis and investigation.

As stated in Part 1 (Ciolino et al., 2021), the CDC concluded that the presence of the additive vitamin E acetate (VEA) in vaping liquid products was strongly linked to the EVALI outbreak (Blount et al., 2020; US Centers for Disease Control, 2020). While VEA was of particular concern, the CDC also concluded that the contribution of other chemicals in the vaping liquid products could not be ruled out in some EVALI cases (US Centers for Disease Control, 2020). Hence, in this second part, we will present results obtained for the mass spectrometric identification of numerous vaping liquid additives from this same subset of vaping liquids.

The first report of vitamin E acetate (VEA) being found as a major component in vaping liquid products used by EVALI patients was from New York (Duffy et al., 2020). Medium chain triglycerides oil (MCT oil) was also identified in many of the EVALI patient vaping fluids, alone or in combination with the VEA. The vaping liquid products were judged to originate from illicit sources (Duffy et al., 2020). There have also been reports (Duffy et al., 2020; Meehan-Atrash and Strongin, 2020) of the direct analysis of liquid additives intended for use in the formulation of Cannabis-based vaping fluids. Analysis of three “commercial diluents” (Duffy et al., 2020) identified VEA only in two of the diluents, and a combination of squalane, MCT oil, and a minor amount of triethyl citrate in the third diluent. Analysis of three “commercial thickeners” (Duffy et al., 2020) identified VEA, squalane, or the terpene α-bisabolol, respectively. Analysis of two additives (Meehan-Atrash and Strongin, 2020) described as “adulterants” obtained from a “cannabis industry source” showed one additive to be pure VEA, and the other additive to contain a pine rosin material in combination with MCT oil.

In addition to these prior reports of vaping liquid additives, a review of the patent literature shows that additives play a major role in the formulation of commercial Cannabis vaping liquids. Commercial vaping liquid additives may include solubilizers/carriers (Zumpano, 2020, Goldman et al., 2015), viscosity modifiers (Finley et al., 2016; Finley and McKee, 2019), flavoring or aroma compounds (Goldman et al., 2015; Finley and McKee, 2019; Green et al., 2020; Zumpano, 2020), terpenes (Tucker and Fulton, 2017), and other additive types (Kotra et al., 2020). A given additive may serve more than one purpose. The EVALI outbreak demonstrates the need for analytical methods which rapidly identify vaping liquid additives in order to provide the most complete information when assessing chemical exposures which may occur during vaping.

This report will provide a summary of the additives identified in the neat vaping liquids, and details of the GC-MS analytical methods used for their identification. Confirmation of selected additives using liquid chromatography-high resolution mass spectrometry (LC-HRMS) will also be presented. The additives include VEA, medium chain triglycerides oil (MCT oil), polyethylene glycols (PEGs), squalane, triethyl citrate, dipropylene glycol dibenzoate (DPG dibenzoate), pine rosin acids, pine rosin methyl esters, and sucrose acetate isobutyrate (SAIB). The peculiarities of identifying several of the more complex additives will be addressed. As analysis was conducted on the neat vaping liquids, this work is not intended to be a vaping study, nor to identify components of aerosols generated during the vaping process.

## Experimental

### Standards, Solvents, and Reagents

Vitamin E acetate (alpha-tocopherol acetate, ≥98%) was obtained from Sigma-Aldrich (St. Louis, MO) or USP (Rockville, MD). Pimaric acid (≥98%) was obtained from Cayman Chemical Company (Ann Arbor, MI). A partially hydrogenated methyl ester rosinate standard material (a complex mixture of methyl esters of rosin acids including both methyl dihydroabietate and methyl dehydroabietate, purity not declared), trioctanoin (≥93%), and tridecanoin (≥98%) were obtained from TCI America. All of the remaining standards or standard materials were obtained from Sigma-Aldrich as follows: abietic acid (technical grade, 75%), dehydroabietic acid (≥95%), isopimaric acid (≥98%), dipropylene glycol dibenzoate (technical grade, 75%), gum rosin (acid value 165), sucrose acetate isobutyrate (food grade), squalane (96%), triethyl citrate (≥98%), and PEG oligomers [tetraethylene glycol (99%), pentaethylene glycol (98%), hexaethylene glycol (97%), octaethylene glycol (≥95%)].

Ethanol (200 proof, USP/ACS grade) and N,N-dimethylformamide dimethyl acetal (GC derivatization grade) were obtained from Sigma Aldrich (St. Louis, MO). Acetonitrile (HPLC grade), pyridine (certified ACS), and formic acid (99+%) were obtained from Fisher Scientific (Waltham, MA). Deionized water (18 Ω) was obtained from a Millipore filtration system fed by a service deionized water source. BSTFA reagent [99:1 N,O-bis(trimethylsilyl)trifluoroacetamide: trimethylchlorosilane] was obtained from Regis Technologies (Morton Grove, IL).

### MCT Oil Reference Sample

Mixed octanoyl/decanoyl-triglycerides are common components of MCT oils, but we were unable to identify a source for these mixed triglyceride standards. An MCT oil product was purchased from a local grocery store in August 2019, and established for use as an MCT oil reference sample with respect to the mixed triglycerides. The product name was “Nature’s Way Organic MCT oil” with a label claim of “100% potency, medium chain triglycerides.” The reference MCT oil sample was analyzed by our laboratory using both GC-MS and direct analysis in real time high resolution accurate mass spectrometry (DART-HRMS) and consisted of four predominant triglycerides: trioctanoin, tridecanoin, a mixed dioctanoyl/monodecanoyl triglyceride, and a mixed monooctanoyl/didecanoyl triglyceride. Identification of the two mixed octanoyl/decanoyl triglycerides was confirmed both by the GC-MS EI spectra and the DART-HRMS accurate mass determinations and spectra. The HRMS observed vs. theoretical masses for the ammonium adducts of the mixed triglycerides were as follows: dioctanoyl/monodecanoyl triglyceride [C_29_H_54_O_6_ + NH_4_^+^], m/z 516.4263 observed versus m/z 516.4259 theoretical, mass error +0.77 ppm, and monooctanoyl/didecanoyl triglyceride [C_31_H_58_O_6_ + NH_4_^+^], m/z 544.4575 observed versus m/z 544.4572 theoretical, mass error +0.55 ppm. The positions of the octanoyl- and decanoyl-substituents on the triglyceride backbones for the two mixed triglycerides were not established.

### Standards Preparation

#### Standards and MCT Oil Reference Sample for Parent Compounds Analysis

Standards for GC-MS analysis as the parent compounds were prepared from stock solutions at finished concentrations in acetonitrile in the range 50–300 μg/ml, and analyzed using GC-MS Protocol A. These include vitamin E acetate, trioctanoin, tridecanoin, dipropylene glycol dibenzoate, sucrose acetate isobutyrate, squalane, triethyl citrate, and also included the MCT oil reference sample.

#### Pine Rosin Acid Methyl Esters Standards Analysis

A solution of methyl abietate was prepared at a finished concentration of 280 μg/ml in derivatizing agent by mixing 25 μl of an abietic acid stock solution, 300 μl pyridine, and 300 μl N,N-dimethylformamide dimethyl acetal in a GC vial, causing conversion of the abietic acid to methyl abietate. A standard mix (see also Figure 5) which contained methyl dihydroabietate, methyl dehydroabietate, and methyl abietate was prepared by mixing aliquots of the methyl abietate solution with an aliquot of a stock solution of a partially hydrogenated methyl ester rosinate standard material. The partially hydrogenated methyl ester rosinate standard material contained both methyl dihydroabietate and methyl dehydroabietate as primary components. The standard mix was analyzed using GC-MS Protocol A.

#### Standards for TMS Derivatives Analysis

For preparation of the standard TMS derivatives, an aliquot of a standard stock solution was transferred to a GC vial for derivatization in the same manner as the samples (see GC-MS Protocol B). Standards were prepared at finished concentrations in the derivatizing reagent as follows: PEG oligomers (200–350 μg/ml), pine rosin acids (10–50 μg/ml), and gum rosin standard material (230 μg/ml), and analyzed using GC-MS Protocol B.

### Removal of Vaping Liquids From Vaping Devices

Used and unused vaping cartridges were received. For used cartridges, the remaining vaping liquid amounts ranged from residues to almost full cartridges. Full cartridges contained up to 1 g or 1 ml of vaping liquid. Prior to sampling for analysis, the vaping liquid contents were transferred from the cartridges or vaping devices to 2 ml autosampler glass vials (Water Corp.) for storage as follows. The receiving vial was placed in the bottom of a 15 ml conical bottom centrifuge tube (Falcon brand). A 5 ml plastic disposable pipet tip (Rainin RC-L5000) was placed into the receiving vial with the pipet tip end pointed downward. The vaping cartridge or device was disassembled, and the open end was placed into the top end of the pipet tip so as to allow flow of the vaping liquid out of the device through the pipet tip and into the receiving vial. The entire assembly was then placed in a centrifuge and spun until transfer of the vaping liquid into the receiving vial was complete (3–5 min). An IEC clinical centrifuge (dial setting 3) or Thermo Sorvall Legend RT centrifuge (2,000 rpm) was used. The amount of vaping liquid recovered from unused cartridges was in the range 0.7–1.0 g, and the amount of vaping liquid recovered from used cartridges was in the range 0.002–0.9 g. Based on visual observation of their flow behaviors, the vaping liquids we encountered typically consisted of medium to high viscosity liquids.

### Preparation of Sample Concentrates for GC-MS Analysis

Due to the limited sample amounts for many of the vaping liquids, and the difficulty of sampling viscous liquids without considerable waste, an initial concentrated extract of the vaping liquid (referred to as the “sample concentrate”) was prepared in 95% ethanol. Sample concentrates were prepared in 1.0 ml or 4.0 ml glass sample vials, with vaping liquid sample weights typically in the range 10–100 mg. Solvent volumes were typically in the range 0.5–1.0 ml, resulting in finished sample concentrates generally in the range 20–100 mg vaping liquid per ml. After addition of solvent, the sample vial was capped and then briefly warmed on a hot plate as needed to speed dissolution of the vaping liquid (one or 2 min, ≤100°C). After dissolution of the vaping liquid, the sample vial was mixed on a vortexer to produce a homogeneous solution. Once prepared, aliquots of the sample concentrate were taken as described below for GC-MS qualitative analysis of the vaping liquid additives. When sufficient vaping liquid was available, duplicate preparations of sample concentrates were made, and analyzed as described.

### GC-MS Sample Preparation and Analysis

Aliquots of the vaping liquid sample concentrates were taken as described below. Vitamin E acetate (VEA, alpha tocopherol acetate), medium chain triglycerides oil (MCT oil), pine rosin acid methyl esters, triethyl citrate, sucrose acetate isobutyrate (SAIB), dipropylene glycol dibenzoate isomers, and squalane were identified as the parent compounds (see GC-MS protocol A). PEG (polyethylene glycol) oligomers, and pine rosin acids were identified as the trimethylsilyl derivatives (see GC-MS protocol B). After initial identification of additive(s) in a given vaping liquid, confirmation of the additive compound(s) was achieved by concurrent analysis of the vaping liquid with the appropriate standard(s) or reference material(s).

#### GC-MS Protocol A

Vaping liquid sample concentrates were mixed on a vortex mixer prior to taking aliquots for subsequent analysis. A dilution of the vaping liquid sample concentrates in acetonitrile was made directly into a GC vial, with aliquot volumes generally in the range 25–100 μl and a finished volume of ca. 1.0 ml after addition of the acetonitrile. GC–MS analysis was conducted using an Agilent 7890B 70 eV EI GC–MS system with 5977B MS detector. The column was a 30 m Agilent 19091S-433HP 5 MS (5% phenyl) with 0.25 mm internal diameter and 0.25 μm film thickness. Injection volume was 1 μl splitless with an injection port temperature of 250°C. The carrier gas was helium with a flow rate of 0.8 ml/min (constant flow mode). Oven program was as follows: initial temperature 60°C with 0.5 min hold, first ramp 25°C/min to 220°C, hold for 10 min, second ramp 10°C/min to 300°C, with a final hold time of 15 min (run time 39.9 min). Transfer line temperature was 280°C. Solvent delay was 3.5 min, and MS acquisition used full scan mode with mass range 40–600 amu.

#### GC-MS Protocol B

A portion (generally in range 50–200 μl) of the underivatized preparation (see GC-MS protocol A) was transferred to a GC vial for derivatization. The solvent was evaporated under a stream of dry air on a Pierce Reactitherm block (nominal block temperature 70–80°C). 200 μl pyridine and 200 μl BSTFA reagent were added to the vial, the vial was capped, mixed, and incubated for 30 min (70–80°C). GC–MS analysis was conducted using an Agilent 7890B 70 eV EI GC–MS system with 5977B MS detector. The column was a 30 m Restek Rxi-35Sil MS (35% silphenylene) with 0.25 mm internal diameter and 0.25 μm film thickness. Injection volume was 1 μl splitless with an injection port temperature of 250°C. The carrier gas was helium with a flow rate of 0.8 ml/min (constant flow mode). Oven program was as follows: initial temperature 60°C with 0.5 min hold, first ramp 25°C/min to 220°C, hold for 10 min, second ramp 10°C/min to 300°C, with a final hold time of 15 min (run time 39.9 min). Transfer line temperature was 280°C. Solvent delay was 7.0 min, and MS acquisition used full scan mode with mass range 40–600 amu.

#### Processing of GC-MS Chromatograms for Figures

The Agilent GC-MS data files were exported as .CSV files, producing two columns of raw data corresponding to the retention times and mass spectral abundances. The .CSV files were then opened in Microsoft Excel (Excel 2016) and saved as Excel files (.xlsx). The Excel data files were used to produce Excel charts (xy scatter charts with smooth line) corresponding to the original chromatograms. For figures with more than one chromatogram, offsetting of the upper chromatogram in the display was accomplished by adding a constant arbitrary abundance value to the entire abundance data column.

### DART-HRMS Identification of Mixed Octanoyl-Decanoyl Triglycerides in MCT Oil Reference Sample

Approximately 5 mg of the Nature’s Way Organic MCT oil reference sample was dissolved in 1.0 ml of acetonitrile, then further diluted in acetonitrile (100 μl aliquot into final volume 1.0 ml). Acetonitrile was used as the method blank. The sample and blank preparations were sampled and analyzed using DIP-it tips (glass capillaries mounted in plastic). DART-HRMS analysis was conducted using a Thermo Scientific (Bremen, Germany) Q Exactive high resolution mass spectrometer (HRMS) equipped with a DART SVP (standardized voltage and pressure) ionization source, VAPUR interface, and linear rail, on which was mounted a module capable of holding 12 DIP-it tips, all from IonSense. Spectra were acquired with the ion source and mass analyzer operating in positive polarity. The DART SVP source was operated with helium gas at a temperature of 250°C, grid voltage of +300 V, and positioned directly in line with the VAPUR interface inlet at a distance of approximately 8.0 mm, or approximately 4.5 mm from the DIP-it tip during analysis. The mass spectrometer was operated with an inlet capillary temperature of 275°C and S-lens rf level of 80.0 (a.u.). Full scan mass spectra were acquired in profile mode with a nominal resolving power (FWHM at m/z 200) of 17,500 at a nominal rate of 10 Hz, over the range m/z 100–1,000, with an automatic gain control (AGC) value of 10^6^ and maximum injection time of 50 ms. MS/MS spectra of precursor ions (±0.5 Da) were acquired using the same parameters and higher-energy collisional dissociation (HCD) at a collision energy of 30.0 eV. Analysis of the DIP-it tips was performed by moving the linear rail at a rate of 0.5 mm/s.

### LC-HRMS Identification of DPG Dibenzoate Isomers

Approximately 5 mg of the vaping liquid was dissolved in 1.0 ml of acetonitrile, then further diluted in 50/50 acetonitrile/deionized water (10 μl aliquot into final volume 1.0 ml). The DPG dibenzoate reference standard stock solution was diluted in 50/50 acetonitrile/deionized water to yield a ca. 8 μg/ml solution. The method blank comprised 10 μl of acetonitrile with 990 μl of 50/50 acetonitrile/deionized water. LC-HRMS analysis was conducted using a Thermo Scientific UltiMate 3000 liquid chromatograph (LC) coupled to a Q Exactive high resolution mass spectrometer (HRMS). Separation was carried out using a Zorbax Rapid Resolution HD Stablebond C18 column (1.8 μm, 2.1 mm ID × 150 mm length). Gradient elution was performed with initial conditions of 95% deionized water with 0.1% formic acid (A) and 5% acetonitrile with 0.1% formic acid (B), linearly ramped to 95% B in 25 min, then held for 15 min. Mobile phase flow rate was constant at 0.200 ml/min. Each injection was preceded by a 7.0-min equilibration at the initial conditions. The injection volume was 1.0 μl and the column was held at 40°C. The MS was equipped with a heated electrospray ionization (HESI) source operated with sheath gas flow rate of 35 units, auxiliary gas flow rate of 5 units, sweep gas flow rate of 2 units, and heater temperature of 75°C. The spray voltage was +3.25 kV and the probe was held at depth position “C” (the third farthest from the MS inlet of four marked positions). The inlet capillary temperature was 275°C. Full scan mass spectra were acquired in profile mode over the range m/z 120–1,200 with nominal resolving power of 140,000 using an automatic gain control target value of 10^6^. Data-dependent MS/MS spectra were collected in profile mode over a range determined by the precursor m/z value with nominal resolving power of 17,500 using an automatic gain control target value of 10^5^ and higher-energy collisional dissociation (HCD) at a collision energy of 30.0 eV. LC-HRMS data were acquired and analyzed using Xcalibur software from Thermo Scientific.

### LC-HRMS Identification of Sucrose Acetate Isobutyrate Compounds

Approximately 5 mg of the vaping liquid was dissolved in 1.0 ml of acetonitrile, then further diluted in 50/50 acetonitrile/deionized water (10 μl aliquot into final volume 1.0 ml). A stock solution of the SAIB standard material (ca. 4 mg/ml) was prepared in 95% ethanol, then further diluted in 50/50 acetonitrile/deionized water to yield a ca. 20 μg/ml solution. The method blank comprised 10 μl of acetonitrile with 990 μl of 50/50 acetonitrile/deionized water. LC-HRMS analysis was conducted using an Agilent 1260 Infinity liquid chromatograph (LC) coupled to a Thermo Scientific Exactive high resolution mass spectrometer (HRMS). Separation was carried out using a Phenomenex Luna phenyl-hexyl column (3.0 μm, 2.0 mm ID × 150 mm length). Mobile phase flow rate was constant at 0.200 ml/min. Gradient elution was performed with initial conditions of 90% deionized water with 0.1% formic acid (A) and 10% acetonitrile with 0.1% formic acid (B), linearly ramped to 95% B in 20 min, then held for 10 min. Each injection was followed by a 7.0 min equilibration at the initial conditions. The injection volume was 1.0 μl and the column was held at 40°C. The MS was equipped with a heated electrospray ionization (HESI) source operated with sheath gas flow rate of 35 units, auxiliary gas flow rate of 5 units, sweep gas flow rate of 2 units, and heater temperature of 125°C. The spray voltage was +3.25 kV and the probe was held at depth position “C” (the third farthest from the MS inlet of four marked positions). The inlet capillary temperature was 275°C. Full scan mass spectra were acquired in profile mode over the range m/z 100–2,000 with nominal resolving power of 100,000 using an automatic gain control target value of 500,000. All-ion fragmentation (AIF) spectra were collected in profile mode over the range m/z 60–1,200 with nominal resolving power of 25,000 using an automatic gain control target value of 1,000,000 and higher-energy collisional dissociation (HCD) at a collision energy of 40.0 eV. LC-HRMS data were acquired and analyzed using Xcalibur software from Thermo Scientific.

## Results and Discussion

It is important to emphasize that this work is not intended to be a vaping study, nor to identify components of aerosols generated during the vaping process. Rather, as analysis was conducted on the neat vaping liquids, this work is intended to provide information on the vaping liquid formulations. Obviously, for a complete understanding and assessment of the hazards associated with vaping products, it is important to determine the compositions of the neat vaping liquids, as well as conduct studies of the aerosols generated during vaping. The following paragraph provides some perspective on the volatility range which is covered by the current GC-MS methods for the identification of additives in the neat vaping liquids.

Based on visual observation of their flow characteristics, the majority of the vaping liquid additives which were identified consisted of low to medium viscosity liquids under ambient conditions. GC-MS is ideal for the analysis of these types of additives because they are sufficiently volatile for analysis as the parent compounds, or suitable for analysis as the trimethylsilyl derivatives. The oven temperature program used in GC-MS protocol A provided good retention and resolution for additives analyzed as the parent compounds (VEA, MCT oil, pine rosin acid methyl esters, triethyl citrate, sucrose acetate isobutyrate dipropylene glycol dibenzoate isomers, squalane), and also provided resolution from the parent cannabinoids. The oven temperature program used in GC-MS protocol B corresponds to the conditions used in our validated method for analysis of the cannabinoids as the trimethylsilyl derivatives (Ciolino et al., 2018). GC-MS protocol B provided good retention and resolution of the additives analyzed as the trimethylsilyl derivatives (PEG oligomers and pine rosin acids), as well as resolution from the cannabinoid trimethylsilyl derivatives. In our current work with GC-MS protocol A, we established that the less volatile terpenes such as d-limonene, linalool, α-terpineol, caryophyllene, and α-bisabolol elute after the solvent delay. We also established that propylene glycol and glycerin, which are common in nicotine vaping liquids, could be detected as either the parent compounds (GC-MS protocol A) or trimethylsilyl derivatives (GC-MS protocol B, with adjustment of the solvent delay to 3.0 min). While we generally noted if any terpenes were detected in our casework, terpenes are not the focus of this work. We only encountered glycerin or propylene glycol in a few cases, where it appeared that a vaping device had been used for vaping of both nicotine and cannabis vaping liquids.

GC-MS identification of the vaping liquid additives was based on retention time and mass spectral comparison to standard materials, as well as comparison to an established reference sample for the MCT oils. Only minor shifts in GC-MS retention times were observed for the additive compounds throughout the study period (ca. 12 months). However, GC-MS confirmation of the additive compounds was achieved by concurrent analysis of the vaping liquid with the appropriate standards or reference materials, allowing for same day comparison of sample and standard retention times. All sample vs. standard or reference material retention time correspondence met our specified requirements of less than 2.0% relative difference.

The majority of the GC mass spectra are not presented here as they are well known and available in commercial libraries such as the Wiley-NIST 2010/2014 2011/2017 editions, and Designer Drug 2014 and 2017 editions. Confirmation of several more complex additives using LC-HRMS and/or DART-HRMS was also conducted, with same day comparison of sample and standard retention times for the LC-HRMS work. Both GC-MS and LC-HRMS spectra are presented for the multicomponent additive sucrose acetate isobutyrate (SAIB), as spectra for the SAIB components were not found in the commercial libraries. In the sub sections which follow, we provide additional references addressing the commercial use for each of the identified additives in Cannabis vaping liquids.

### Single Component Additives (VEA, Triethyl Citrate, Trioctanoin, Squalane)

**FIGURE 1:**
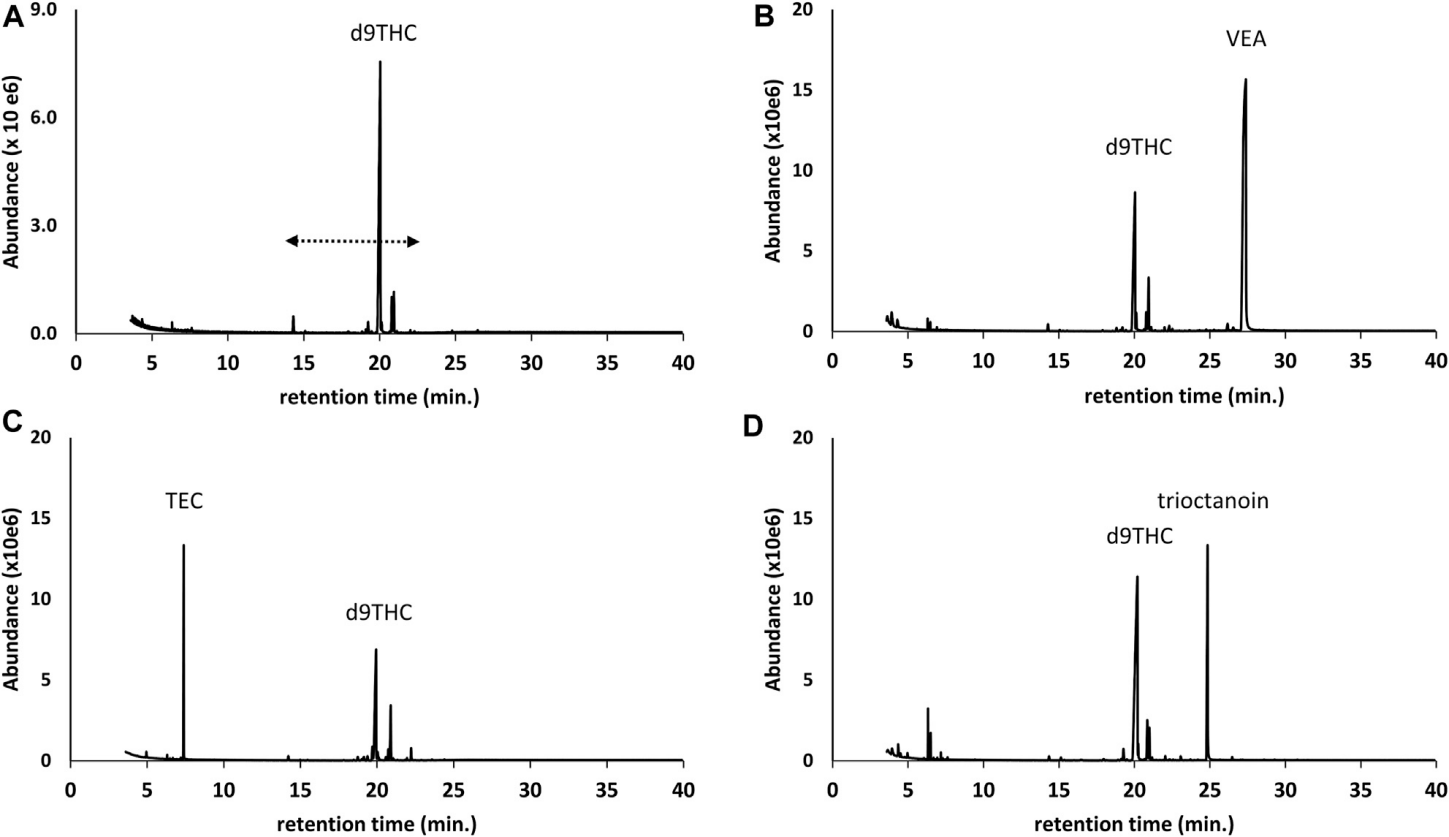
GC-MS chromatograms for d9THC vaping liquids in which no additives were identified **(A)**, and for which single compound additives were identified as follows: vitamin E acetate (VEA) **(B)**; triethyl citrate (TEC) **(C)**; and trioctanoin **(D)**. The retention range for the parent cannabinoids is indicated by the dashed double arrow (as shown in Panel 1**A**), and the minor peaks in this range for all chromatograms correspond to other cannabinoids including CBD, CBG, and CBN. The early eluting peaks in Panel 1**(D)** (less than 8 min) correspond to terpenes.

Several of the additives we encountered were single component additives, and include vitamin E acetate (alpha-tocopherol acetate), triethyl citrate, trioctanoin, or squalane. Figure 1B through Figure 1D show chromatograms for underivatized preparations of d9THC vaping liquids in which vitamin E acetate, triethyl citrate, or trioctanoin were identified, respectively. For comparison, Figure 1A shows a chromatogram for a vaping liquid in which no additives were identified and also shows the retention range for the cannabinoids. Identification of these single component additives was straight forward based on retention time and mass spectral correspondence to reference standards. Use of the parent compound alpha-tocopherol (not its acetate ester) as a viscosity modifier, to inhibit oxidation, and to increase bioavailabity in commercial Cannabis vaping liquids has been reported (Finley et al., 2016; Finley and McKee, 2019). The use of triethyl citrate as a lipid solubilizer (Goldman et al., 2015), and the use of trioctanoin as a carrier liquid (Zumpano, 2019), in commercial Cannabis vaping liquids have also been reported.

### Multi-Component Additives

Several complex or multi-component additives were encountered in the vaping liquids. These include MCT oils, PEG oligomers, pine rosin acids, pine rosin derived methyl esters, dipropylene glycol dibenzoate (DPG dibenzoate) isomers, and sucrose acetate isobutyrate (SAIB) compounds. Unlike the single component additives, identification of these more complex additives presented more challenges including some difficulties in obtaining standards or reference materials, and the GC-MS spectra were either not included in commercial libraries or overall less definitive for identification purposes. Additional analysis was conducted using LC-HRMS analysis for the DPG dibenzoate isomers and the SAIB compounds. In the discussion which follows, a brief description is given for each additive, including prior reports of their use in Cannabis vaping liquids.

#### MCT Oils

MCT oils are generally derived from natural oils which have high native contents of medium chain triglycerides, such as coconut oil. The multi-step manufacturing process involves hydrolysis of the glycerides (mono, di, and tri) from the natural oil to produce the fatty acids, then taking the desired medium chain cut of fatty acids, typically in the C6–C12 range. The fatty acids are then reesterified with glycerin, generally to a full extent, producing the triglycerides which make up the MCT oil. The finished MCT oils are low viscosity liquids. The use of medium chain triglyceride oils (MCT oils) as carrier liquids in commercial Cannabis vaping liquids has been reported (Zumpano, 2019).

**FIGURE 2:**
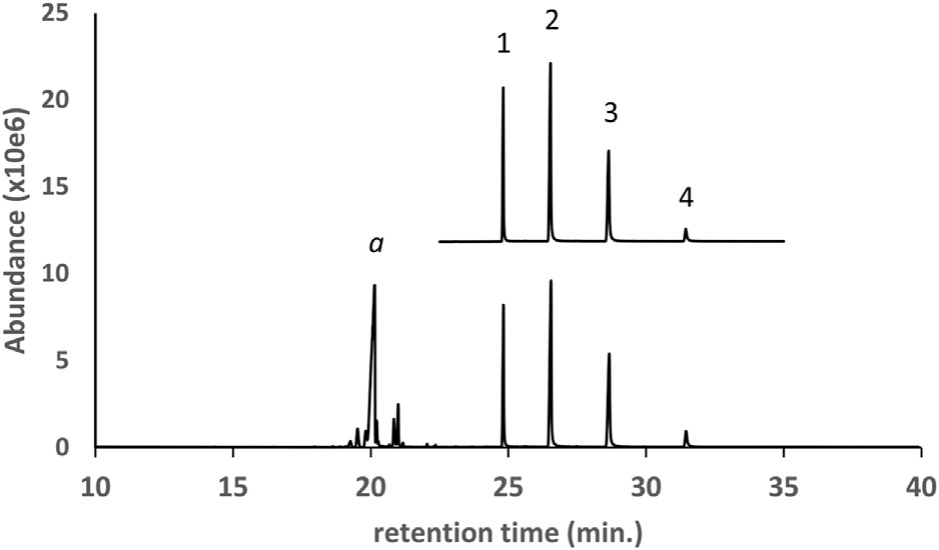
GC-MS chromatogram for a d9THC vaping liquid (peak label a, **lower trace**) in which medium chain triglycerides (MCTs) were identified. The established MCT oil reference sample (**upper trace**, expanded scale, offset) is shown for comparison. The triglycerides were identified as follows: 1- trioctanoin; 2- dioctanoyl/monodecanoyl triglyceride; 3- monooctanoyl/didecanoyl triglyceride; and 4- tridecanoin.

Figure 2 shows the GC-MS chromatogram for a d9THC vaping liquid (lower trace) in which medium chain triglycerides (MCTs) were identified. The locally purchased MCT oil reference sample (upper trace, expanded scale, offset) is shown for comparison. Four triglycerides (peak labels 1–4, respectively) were identified as trioctanoin, an dioctanoyl/monodecanoyl triglyceride, a monooctanoyl/didecanoyl triglyceride, and tridecanoin. The trioctanoin and tridecanoin were confirmed with reference standards, and the two mixed octanoyl/decanoyl-triglycerides were confirmed by comparison with the established MCT oil reference sample. For MCT oils in vaping liquids, this example represents the most frequent pattern of triglycerides we encountered with higher levels of the first three triglycerides and lower levels of tridecanoin. Other minor triglycerides encountered in some of the vaping liquids included hexanoin, dodecanoin, and other mixed chain length triglycerides. Some mono- and diglycerides were occasionally seen.

#### PEG Oligomers

Polyethylene glycols (PEGs) are synthetic oligomers/polymers of ethylene oxide which are manufactured over a wide range of chain length/molecular weights. A given PEG is typically composed of a distribution of chain lengths/molecular weights around an average value, such as PEG 400 with an average molecular weight of 400. PEGs such as PEG 200 and 400 are liquid under ambient conditions, while higher molecular weight PEGs with average molecular weights above 600 are typically solids. The use of PEG 300 and/or 400 as emulsifiers or cosolvents in commercial Cannabis eliquid or vaping formulations has been described (Llamas, 2015; Eck and Pelloni, 2020).

**FIGURE 3:**
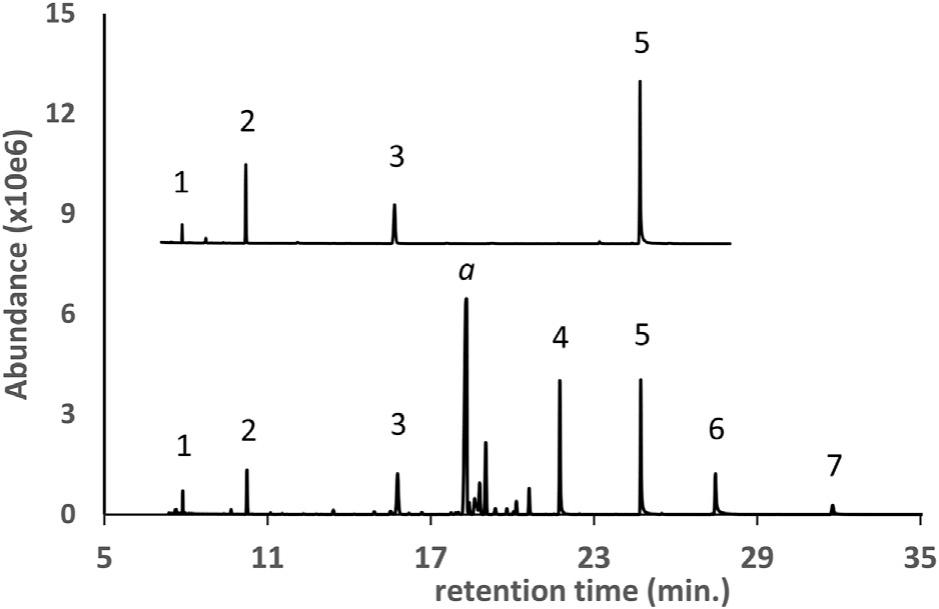
GC-MS chromatogram for a d9THC vaping liquid (peak label a, **lower trace**) in which a series of polyethylene glycol oligomers (PEG oligomers, peak labels 1 through 7) were identified as the diTMS derivatives. PEG oligomer standard mix (**upper trace**, condensed scale, offset) as follows: 1- tetraethylene glycol; 2- pentaethylene glycol; 3- hexaethylene glycol; 5- octaethylene glycol.

The GC-MS chromatogram for a vaping liquid in which polyethylene glycol oligomers (PEG oligomers, peak labels 1–7) were identified is shown in Figure 3 (lower trace). While smaller PEGs may be volatile enough for analysis without derivatization, conversion to the diTMS derivatives prior to analysis provides significantly better peak shape and greatly increased signal response. A standard mix of the diTMS derivatives of tetraethylene glycol, pentaethylene glycol, hexaethylene glycol, and octaethylene glycol (Figure 3, upper trace, peak labels 1, 2, 3, and 5 respectively) is shown for comparison. The additional PEG oligomers (peak labels 4, 6, and 7) are presumed to be the hepta-, nona-, and decaethylene glycols for which standards were not available. While the GC-MS spectra of the PEG oligomer diTMS derivatives are very similar allowing assignment as PEGs, the spectra do not exhibit molecular or high mass ions which would allow an assignment of molecular weight. Our laboratory was able to confirm the PEG oligomer exact masses and identities using DART-HRMS (data not presented in this work).

#### Pine Gum Rosin Acids

**FIGURE 4:**
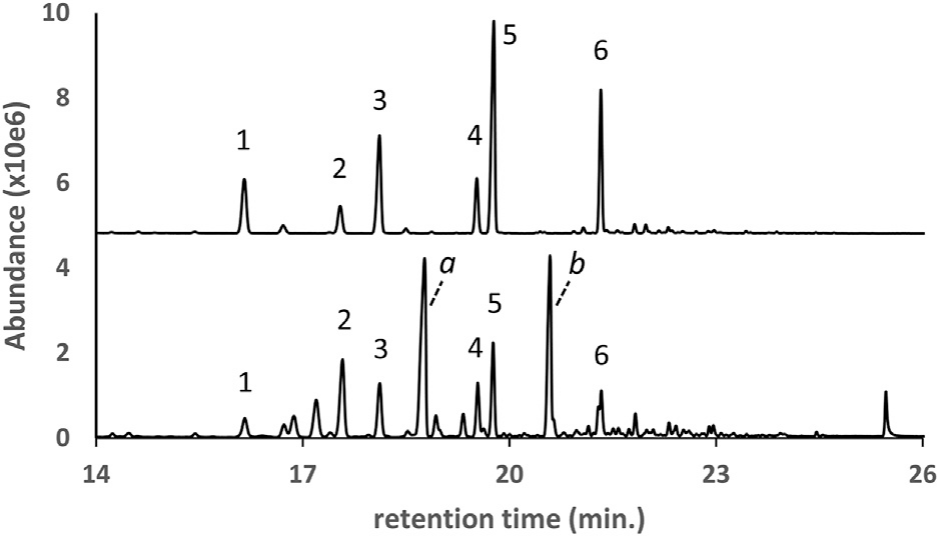
GC-MS chromatogram for a vaping liquid (**lower trace**) in which a series of pine rosin acids (peak labels 1 through 6) were identified as the monoTMS derivatives. A gum rosin standard material (**upper trace**, condensed scale, offset) is shown for comparison. Specific pine rosin acids identified as follows: 2- isopimaric acid; 4- dehydroabietic acid; 5- abietic acid. The vaping liquid contained predominant amounts of d9THC and CBN (peak labels a and b), as well as MCT oil components (not shown).

Pine gum rosin (colophony) is derived from pine tree trunks and is a solid, resinous material which is largely nonvolatile and chiefly composed of a series of related diterpenoid acids such as abietic acid (Organic Materials Review Institute, 2014). The GC-MS chromatogram for a d9THC vaping liquid in which pine rosin acids were identified as the monoTMS derivatives is shown in Figure 4 (lower trace). A derivatized preparation of a gum rosin standard material (Figure 4, upper trace), is shown for comparison. A series of six rosin acids were found in common between the vaping liquid and the gum rosin standard material including abietic acid, dehydroabietic acid and isopimaric acid (peak labels 5, 4, and 2, respectively). While standards were not available to confirm the identity of the other three rosin acids (peak labels 1, 3, and 6), the GC-MS retention times and mass spectra were consistent between the vaping liquid and the gum rosin standard material. In addition to the pine rosin material, the vaping liquid also contained MCT oil components. Note that the combination of pine rosin and MCT oil components was reported in the prior analysis of a commercial “adulterant or additive formulation” (Meehan-Atrash and Strongin, 2020).

#### Partially Hydrogenated Pine Rosin Acid Methyl Esters

Processing of pine rosin for industrial uses may include partial or full hydrogenation followed by esterification (Eastman Chemical Company, 2017). Natural acids found in pine rosin include abietic acid and dehydroabietic acid, for which the chemical structures contain two or three double bonds, respectively. Abietic acid may be partially hydrogenated to form dihydroabietic acid, which contains only one double bond (Eastman Chemical Company, 2017). Esterification with methanol produces the methyl esters. The finished “methyl ester of hydrogenated rosin” materials are viscous liquids. The use of “methyl ester of partially hydrogenated rosin” as an additive in commercial Cannabis vaping compositions has been described (Cameron et al., 2019).

**FIGURE 5:**
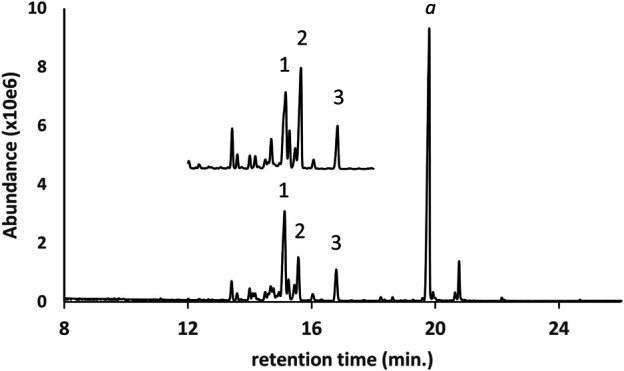
GC-MS chromatogram for a d9THC vaping liquid (peak label a, **lower trace**) in which a series of pine rosin acid methyl esters were identified, compared to a standard mix (**upper trace**, offset, condensed scale) of a partially hydrogenated methyl ester rosinate reference material with a methyl abietate standard. The predominant esters were identified as methyl dihydroabietate, methyl dehydroabietate, and methyl abietate (peak labels 1–3, respectively).

The GC-MS chromatogram for a d9THC vaping liquid in which both natural and partially hydrogenated pine rosin acid methyl esters were identified is shown in Figure 5 (lower trace). The most abundant pine rosin derived methyl esters in the vaping liquid were methyl dihdyroabietate, methyl dehydroabietate, and methyl abietate (peak labels 1–3, respectively). A standard mixture which contained these same three rosin esters was analyzed for comparison (upper trace). The standard mixture was prepared from a partially hydrogenated methyl ester rosinate reference material and a methyl abietate standard (see *Materials and Methods* section). Several additional pine rosin methyl esters in the sample chromatogram were not confirmed with standards but the retention times and spectra were consistent with the standard mixture.

#### Dipropylene Glycol Dibenzoate Isomers

Dipropylene glycol dibenzoate (DPG dibenzoate) is a viscous liquid which may be derived from the esterification of dipropylene glycol with benzoic acid (Zheng et al., 2016), or the transesterification of methylbenzoate with dipropylene glycol (Hulsmann and Renckhoff, 1978). The dipropylene glycol starting material typically comprises multiple isomers (Sexton and Britton, 1953; Hulsmann and Renckhoff, 1978), which include the diprimary alcohol, the disecondary alcohol, and the primary-secondary alcohol. As such, the finished dibenzoate ester is also a mixture of isomers. While DPG dibenzoate is used as an industrial plasticizer (Zheng et al., 2016), our literature search found no references for the use of DPG dibenzoate in vaping liquids.

**FIGURE 6:**
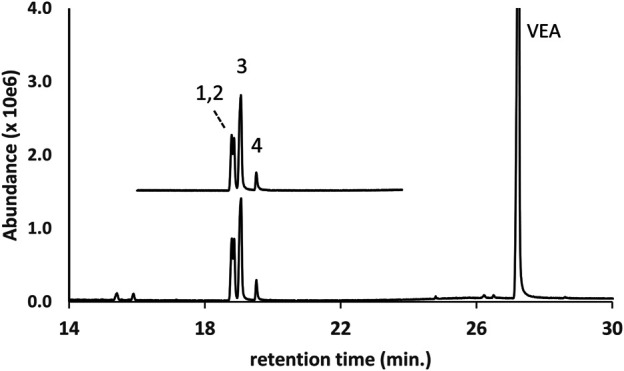
GC-MS chromatogram for a vaping liquid (**lower trace**) in which multiple DPG dibenzoate isomers (peak labels 1–4) were identified in addition to VEA (VEA peak offscale). The DPG dibenzoate standard chromatogram (**upper trace**, offset) shows a similar distribution of the four isomers. The vaping liquid contained only a trace of d9THC (peak not visible on current scale).

The GC-MS chromatogram for a vaping liquid in which four DPG dibenzoate isomers were identified as the parent compounds is shown in Figure 6 (lower trace, peak labels 1–4). The DPG dibenzoate standard chromatogram (upper trace, offset) is shown for comparison, and shows a similar distribution of isomers. The vaping liquid also contained a high content of VEA with only a trace of d9THC (less than 0.1% w/w). The presence of multiple DPG dibenzoate isomers in this vaping liquid was confirmed using LC-HRMS, with exact mass confirmation of the molecular formula C_20_H_22_O_5_ for the DPG dibenzoate isomers (calculated mass error of 1.17 ppm for the H^+^ adduct of the predominant isomer, m/z 343.1544 versus theoretical m/z value of 343.1540).

#### Sucrose Acetate Isobutyrate Compounds

**FIGURE 7:**
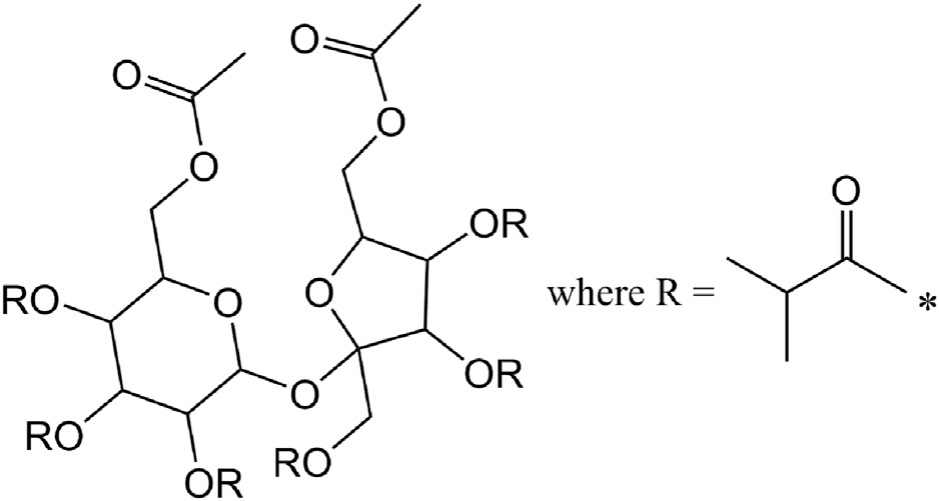
Common representation of SAIB as sucrose diacetate hexaisobutyrate ignoring the variations in the numbers of acetate and isobutyrate moieties and their positions.

Sucrose acetate isobutyrate (SAIB, sucrose diacetate hexaisobutyrate) is a viscous liquid which is generally derived from the esterification of sucrose with acetic and isobutyric anhydrides (Goins and Davis, 1963). Because sucrose has a total of eight reactive hydroxy sites and the synthesis is typically conducted with a molar ratio of 2:6 acetic:isobutyric anhydrides, SAIB is usually depicted as the diacetate hexaisobutyrate (Figure 7) with a corresponding unit mass of 846 g/mol. However, as our data will show, the esterification actually produces a highly complex mixture of SAIB compounds with varying numbers of acetate and isobutyrate ester substituents per sucrose molecule, as well as variations in the positions of the ester moieties. The use of SAIB as a weighting agent in commercial Cannabis vaping liquids (Goldman et al., 2015) has been reported.

**FIGURE 8:**
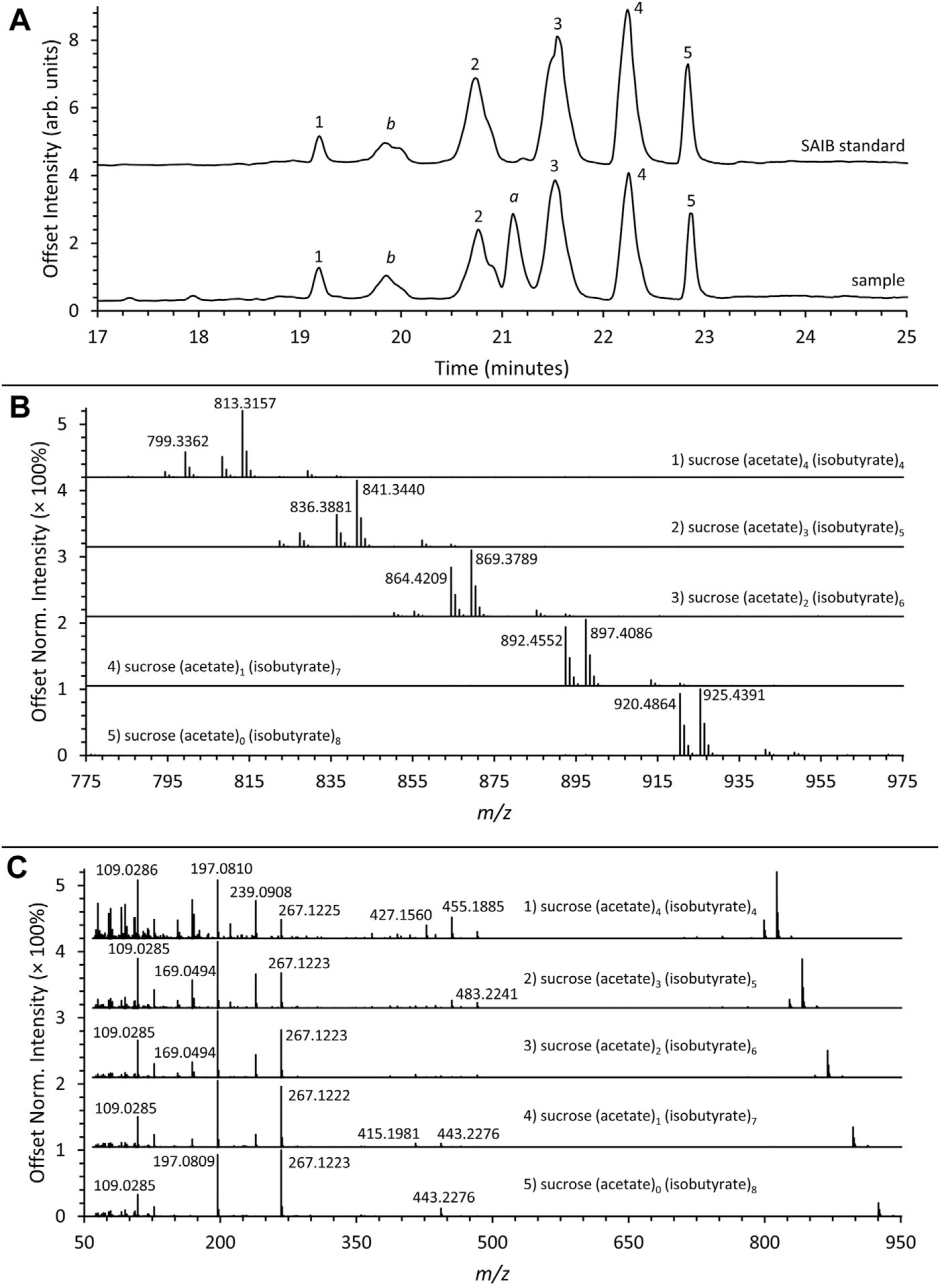
**(A)** LC-HRMS total ion chromatograms of the SAIB reference standard **(top)** and sample **(bottom)** showing a series of five SAIB peaks (peak labels 1–5). Peak a corresponds to d9THC and peak b corresponds to a background impurity also present in blanks. **(B)** Mass spectra corresponding to the five SAIB chromatographic peaks. **(C)** AIF spectra corresponding to the five SAIB chromatographic peaks.

A vaping liquid containing a series of SAIB compounds was analyzed using both LC-HRMS and GC-MS. The LC-HRMS total ion chromatogram for the vaping liquid is given in Figure 8A (lower trace) with comparison to the SAIB standard material (upper trace). Similar profiles for a series of five SAIB peaks (retention times ∼19.9, 20.7, 21.5, 22.2, and 22.8 min) were observed for both the vaping liquid and standard material. The HRMS spectra for the five peaks (Figure 8B) showed the presence of both Na^+^ (M + 23) and NH_4_^+^ (M + 18) adducts, with higher intensities observed for the Na^+^ adducts. The Na^+^ adduct exact masses for the five peaks correspond to SAIB compounds containing varying number of acetate and isobutyrate substituents as listed in Table 1, first four columns. It is assumed that each of the first four SAIB peaks include coeluting SAIB compounds with the same number of acetates and butyrates per sucrose molecule, but in differing positions. The molecular weight for the fifth and last peak (902 g/mol) corresponds to the octaisobutyrate compound.

**TABLE 1:** LC-HRMS exact mass groupings of SAIB compounds and corresponding GC-MS high mass ion fragments.

SAIB compounds exact mass (molecular formula)	SAIB Na^+^ adduct LC-HRMS exact mass theoretical/measured (Δ ppm)	# Acetates/molecule	# Isobutyrates/molecule	GC-MS SAIB compounds unit mass	# Acetates/fragment	# Isobutyrates/fragment	GC-MS high mass fragment unit mass
790.3259 (C_36_H_54_O_19_)	813.3157/813.3157 (0.0 ppm)	4	4	790	4	0	331
					3	1	359
					2	2	387
					1	3	415
					0	4	443
818.3572 (C_38_H_58_O_19_)	841.3470/841.3440 (−3.6 ppm)	3	5	818	3	1	359
					2	2	387
					1	3	415
					0	4	443
846.3885 (C_40_H_62_O_19_)	869.3783/869.3789 (0.7 ppm)	2	6	846	2	2	387
					1	3	415
					0	4	443
874.4198 (C_42_H_66_O_19_)	897.4096/897.4086 (−1.1 ppm)	1	7	874	1	3	415
					0	4	443
902.4511 (C_44_H_70_O_19_)	925.4409/925.4391 (−1.9 ppm)	0	8	902	0	4	443

Molecular ions for the SAIB compounds are not observed in EI GC-MS analysis, but the high mass ion fragments corresponding to the various tetraester distributions on the glucose and fructose fragments are observed. See text for discussion.

**FIGURE 9:**
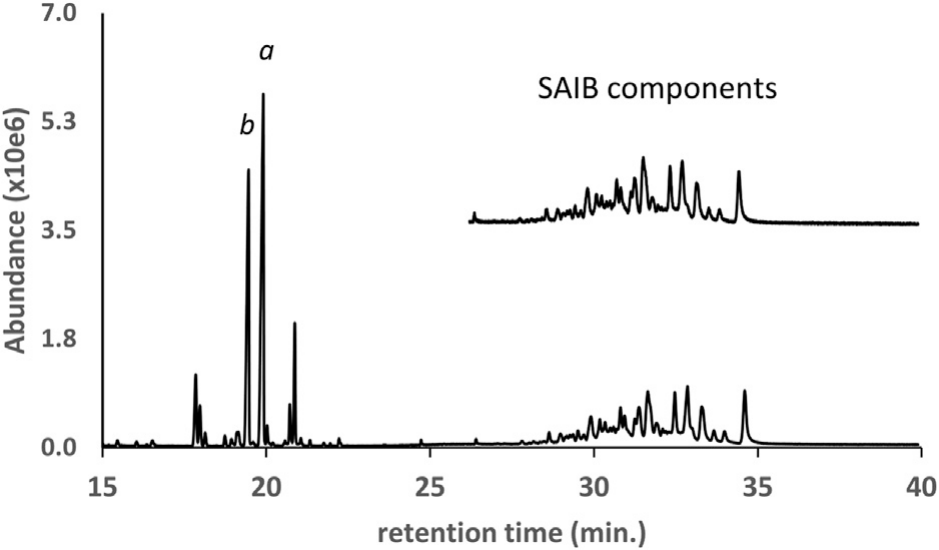
GC-MS chromatogram for a vaping liquid (lower trace) in which a series of sucrose acetate isobutyrate (SAIB) compounds were identified (retention time range 28–35 min). The SAIB standard chromatogram (upper trace, expanded scale, offset) shows a similar distribution of components. The vaping liquid contained substantial levels of both d9THC and d8THC (peak labels a and b, respectively).

**FIGURE 10:**
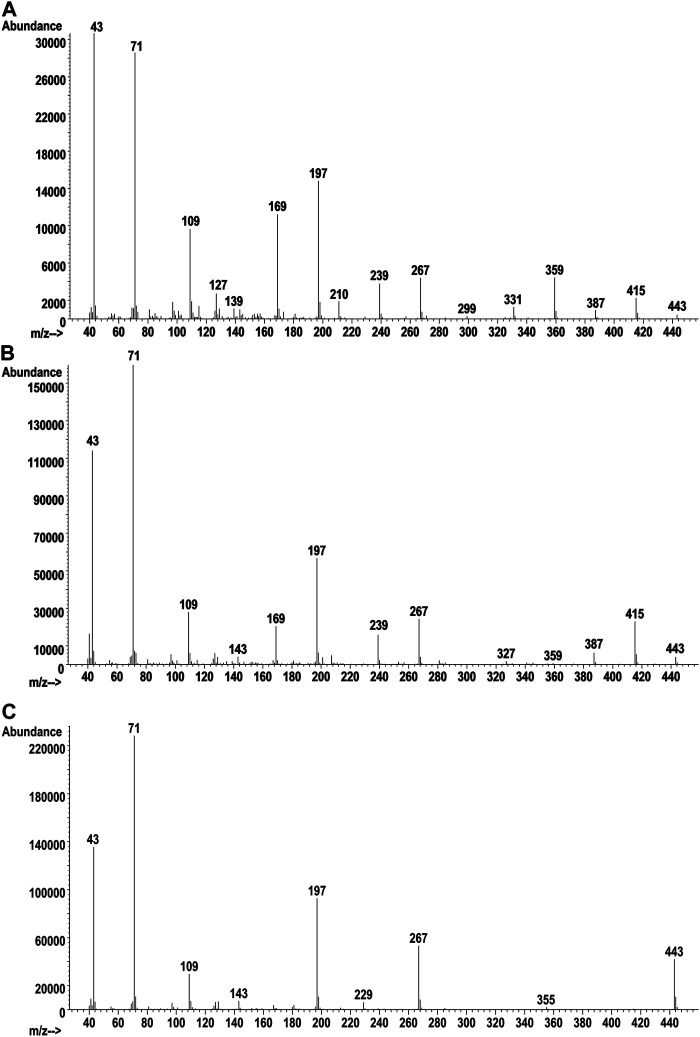
Vaping liquid SAIB component GC-MS spectra for earliest eluting peak (**A**, 28.6 min), mid eluting peak (**B**, 31.6 min), and last eluting peak (**C**, 34.4 min).

The GC-MS chromatogram for the same vaping liquid is given in Figure 9 and shows a series of more than 15 closely spaced SAIB components (lower trace, retention range 28–35 min). Again, a very similar distribution of the SAIB components was observed in comparison with the SAIB standard material (Figure 9, upper trace). GC-MS mass spectra for three representative SAIB components (retention times 28.6, 31.6, and 34.4 min) are given in Figure 10. In the GC-MS spectra for sucrose esters including SAIB (Severson et al., 1985; Uematsu et al., 2001), molecular ions are not observed under EI conditions. Rather, the high mass ions in the EI spectra represent the esterified glucose or fructose fragments minus the bridge oxygen, which is transferred to the neutral fragments (Severson et al., 1985). Cleavage at the oxygen bridge may occur on either side such that both ionized glucose and fructose ion fragments are formed. In the case of SAIB, these high mass ions correspond to glucose and/or fructose tetraesters with varying numbers of acetate and isobutyrate moieties.

Table 1 also provides the GC-MS high mass ion fragment unit masses which may be observed for various SAIB positional isomers (see last four columns) within each of the mass groupings from the LC-HRMS analysis. This listing only addresses positional isomers with respect to the numbers of acetates or isobutyrates on a glucose or fructose fragment, but does not address isomeric positions on the monosaccharides. Glucose vs. fructose fragments are also not differentiated in the analysis. Based on molecular weight considerations, the early eluting SAIB compounds are expected to have the least number, and the late eluting compounds the most number, of isobutyrate substituents. The mass spectra are consistent with this expectation, with the earliest eluting peak showing all five of the high mass fragments listed in Table 1 for SAIB compounds with four acetates and four isobutyrates (m/z 331, 359, 387, 415, 443), and the latest eluting peak showing only the high mass fragment for the octaisobutyrrate compound (m/z 443).

Figure 8C shows the all-ion fragmentation (AIF) spectra for the five SAIB peaks from the LC-HRMS work. The lower mass ions at m/z 267, 239, 197, 169, and 109 in both the AIF (Figure 8C) and the GC-MS spectra (Figure 10) have been described (Uematsu et al., 2001). The remaining acetate or isobutyrate moieties on the fragments are as follows: two remaining isobutyrates (m/z 267), one acetate and one isobutyrate (m/z 239), one isobutyrate (m/z 197), one acetate (m/z 169), and no remaining moieties (m/z 109).

Based on visual examination of the GC-MS chromatograms, it is apparent that the additives identified in this work were frequently major constituents in the vaping fluids. Peak heights for single component additives (Figures 1B–D), or cumulative peak heights for multicomponent additives (Figures 2–6, and 9), may be comparable to or exceed the peak height of the vaping liquid active constituent, such as Δ^9^-tetrahydrocannabinol. In other cases, only minor amounts of additives were found. Formal quantitation of the additives in the vaping liquids was only conducted for VEA and the MCT oils, and was conducted using GC-FID methods which were developed and validated within our laboratory (Lanzarotta, 2020). VEA levels ranging from 4–88 %w/w were determined in a grouping of 127 vaping liquids, with a grand average of 50% w/w (Lanzarotta, 2020). MCT oil levels ranging from 0.2 to 66 %w/w were determined from a grouping of 55 vaping liquids, with a grand average of 15% w/w (Lanzarotta, 2020). While there is significant overlap among the ca. 300 vaping liquids reported in this study and the vaping liquids subjected to quantitative analysis for VEA and the MCT oils, the quantitative data summaries were conducted independently by the analysts conducting the quantitative work.

## Summary of Findings

**FIGURE 11:**
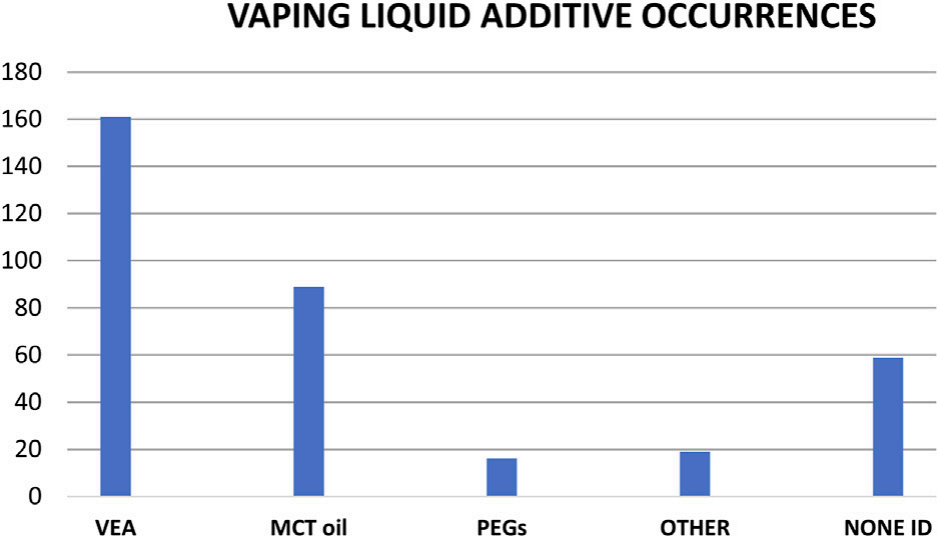
Number of vaping liquid additives occurrences in a composite grouping of ca. 300 vaping liquids representing both EVALI patient associated and independent investigations. See text for discussion.

Approximately half of the ca. 300 vaping liquids from this study were associated with EVALI patients, and the other half were obtained in the United States through independent investigations in the time frame after the EVALI outbreak (late 2019 and early 2020). The additive findings between both groups of vaping liquids were generally consistent, and are summarized in Figure 11 for the various additives. Note that Figure 11 shows the composite findings for the entire group of ca. 300 vaping liquids, representing vaping liquids from both EVALI patient associated and independent investigations. No additives were identified in approximately 20% of the vaping liquids, and VEA was found in 50–60% of the vaping liquids. On average, MCT oils were found in 30% of the vaping liquids, and PEGs in about 5% of the liquids.

We found multiple additives in about 60 of the vaping liquids, with the most frequent combination being VEA and MCT oil. Other combinations included MCT with SAIB, MCT with PEG, MCT with pine rosin acids, and VEA with DPG dibenzoate. A few vaping liquids contained the three additives VEA, MCT oil, and SAIB. In addition to the additives in this report, we found terpenes in about 85% of the vaping liquids. We also saw flavoring components in some vaping liquids, and indications of various vegetable oils. Given the explosion of technological development in the commercial Cannabis industry, analytical and forensic laboratories are likely to encounter even more additive types in products such as vaping liquids. GC-MS analysis is an ideal approach for identification of a wide variety of vaping liquid additives, and LC-HRMS with exact mass determination provides definitive confirmation of identity.

This summary of findings is intended to provide information which is useful to analytical, forensic, or other testing laboratories who may conduct testing of cannabis based vaping liquids. The data sets presented here, and the summary of findings, are not intended to represent an epidemiological evaluation linked with the EVALI outbreak.

## Data Availability

The datasets presented in this article are not readily available because data may be considered sensitive or require a special request for public disclosure. Requests to access the datasets should be directed to laura.ciolino@fda.hhs.gov.
